# Real world data analysis of next generation sequencing and protein expression in metastatic breast cancer patients

**DOI:** 10.1038/s41598-020-67393-9

**Published:** 2020-06-26

**Authors:** Dirk Hempel, Florian Ebner, Arun Garg, Zeljka Trepotec, Armin Both, Werner Stein, Andreas Gaumann, Lucia Güttler, Wolfgang Janni, Amelie DeGregorio, Louisa Hempel, Valeria Milani

**Affiliations:** 1 Cancer Center Dachau-Donauwoerth/Steinbeishochschule, Berlin, Germany; 2 O&G Department, Cancer Center Dachau, Dachau/Universität Ulm, Krankenhausstr. 15, 85221 Dachau, Germany; 3 Donauauries Klinik Donauwoerth, Donauwörth, Germany; 4 Pathologie Suedbayern, Penzberg, Germany; 5 Facharztzentrum Fuerstenfeldbruck, Fürstenfeldbruck, Germany; 6 Frauenklinik der Universität Ulm, Ulm, Germany; 7 Sigmund Freud University Vienna, Vienna, Austria

**Keywords:** Breast cancer, Cancer genomics

## Abstract

Next generation sequencing (NGS) together with protein expression analysis is back bone of molecularly targeted therapy in precision medicine. Our retrospective study shows our experience with NGS of 324 genes in combination with protein expression in patients with advanced breast cancer (aBC). The primary purpose was to analyze the prevalence of individual genetic alterations combined with protein expression to define potential targets for an individualized therapy. Between April 2018 and September 2019, 41 patients with aBC were offered a NGS test. The test was used to detect clinically relevant genomic alterations and to support further targeted therapy decisions. Hormone receptors, ERBB2 of tumors and PD-L1 was stained by immunohistochemistry. The data was recorded up to September 2019. After prior consent 41 results were available for further analysis. The most common BC subtypes were triple-negative (n = 16), HR+/ERBB2− (n = 15), and ERBB2+ (n = 9), with one missing data of the primary tumor. 27 patients had more than one genetic alteration. The most common alterations were *PIK3CA *(n = 14) and *ERBB2* alterations (n = 11). Followed by *ESR1 *(n = 10), *FGFR1 *(n = 7) and *PTEN *(n = 7). 68% of the alterations were clinically relevant (tier I and II of ESCAT classification). The most common treatment recommendation was ERBB2-directed therapy (single or double blockade, trastuzumab emtansine and lapatinib) followed by alpelisib in combination with fulvestrant. Comprehensive genomic profiling combined with protein expression analysis in aBC allowed a guided personalized therapy for half of our patients. So far there are no well-defined tools allowing interpretations of genomic alterations detected by NGS in combination with protein expression and other factors.

## Purpose

The traditional risk and treatment assessment of primary breast cancer was traditionally based on tumor size, lymph node involvement, grading and proliferation index according to the Ki67, and hormone receptor and ERBB2 amplification status^1^. The recommendation for treatment strategies in the (neo-)adjuvant setting of breast cancer therapy was improved due to the implementation of multi gene expression tests such as Oncotype DX and EndoPredict tests in selective patients’ cohorts.

Spite of these advances in molecular analysis of primary tumor genotypes, in the setting of advanced disease, molecular genetic-based biomarkers are not used routinely. With the publication of Perou et al., BC was classified according to its molecular profile in intrinsic subtypes^2^. These subtypes differ in their response to systemic treatment and short-/long-term prognosis. Based on these intrinsic subtypes, further understanding of the genetic alterations and pathways has been gained. This increased knowledge has led to the development of therapies targeting special genetic alterations in tumor cells or cells within the microenvironment of a tumor. Alpelisib and Olaparib are two targeted therapies recently approved by the FDA accordingly to the mutational status of tumor. Alpelisib^3,4^ is indicated in HR+/ERBB2− metastatic breast cancer with PIC3CA mutation and Olaparib^5^ is approved for treatment of germline *BRCA* mutated advanced disease.

Germline mutations of *BRCA* gene can be detected in approximately 5% of all breast cancers^5^. These patients benefit from PARP inhibitors like Olaparib^6^. The clinical evidence of somatic *BRCA* mutation in mBC is unclear and could depend from the detected mutant allele fraction (MAF).

So far there are no well-defined tools that allow interpretations of genomic alterations detected by NGS in combination with protein expression of tumor^7^. However, there are different frameworks that assign individual gene alterations and corresponding treatments, classified into tiers by the evidence strength from clinical studies; the most established so far is the ESMO Scale for Clinical Actionability of molecular Targets (ESCAT).

In this explorative analysis we present the data of our advanced BC patients and their treatment options based on a solid tumor genomic profiling in conjunction with protein expression. The applied FoundationOne CDx is based on Illumina platform, that has been approved by the FDA on November 30, 2017^8^.

## Methods

Starting from April 2018, our cancer center has had access to a hybrid capture based NGS service platform (FoundationOne CDx) for solid tumor samples and subsequently offered this service to patients with advanced disease. The test has been FDA approved for breast cancer since 2017. The test was used to detect clinically relevant genomic alterations (point mutations, indels, rearrangements, and CNAs), and to support the selection of an appropriate targeted therapy by the physicians. The assay interrogates 324 genes, as well as introns of 34 genes involved in rearrangements^9^ and does also report Tumor Mutational Burden (TMB)^10^ as well as Microsatellite Instability (MSI)^11^. Results provide a comprehensive molecular tumor profile, as previously described elsewhere^12,13^. For each tumor profile, individual therapy options are provided according to the current state of scientific knowledge and approval.

### Patients characteristics

From April 2018 up to September 2019, a total of 335 samples were analyzed after receiving informed patient consent of patients for scientific purposes with an advanced solid tumor. We collected 41 samples originating from metastatic sites of patients with advanced breast cancer. Histopathological and immunohistochemical examination of sample confirmed the primary diagnosis of breast cancer. The final database included 41 successfully analyzed BC samples harboring alterations and protein expression (HR, ERBB2 in immune cells, PD-L1) by IHC (Addendum A).

Patient´s characteristics including age, primary tumor subtype, grading, site of metastasis and previous therapies were retrospectively analyzed and correlated with genomic alterations and protein expression and potential treatment options. The results of hormonal assessment for ER, PR, and ERBB2 were dichotomized into negative versus positive. PD-L1 status was determined by using Combined Positive Score (CPS) and Tumor Proportion Score (TPS) with different antibody clones (SP263, SP142 and CAL10). Genomic alterations were clustered into belonging signaling pathways and further analyzed by measures of central tendency. Due to the short follow-up interval of 1.5 years (April 2018–September 2019), a clinical follow up after molecular based treatment decision is not reportable at time and not the primary endpoint of study.

## Results

Starting in April 2018 up to September 2019, n = 41, advanced BC patients were offered the solid tumor genomic profiling test FoundationOne CDx. The patient’s characteristics including clinicopathological profile are summarized in Table 1. Our cohort of patients included 100% female with a median age at diagnosis of 50 (range 31–84) and except for one woman, they were all in a postmenopausal status. The most common BC subtypes were triple-negative (n = 16), followed by HR+ (n = 15), and ERBB2+ BC (n = 9). In our cohort, the average number of metastatic sites per patient was more than 2. In four patients, PD-L1 status was positive (> 1% score), with a tumor grading G2 (n = 2) and G3 (n = 2).

**Table 1:** Clinicopathological profile of patients (n = 41).

Characteristics	Number of patients	%
**Age (years)**		
Median	59	
Range	31–84	
**Breast cancer subtype**		
HR+, ERBB2−	15	36.6
ERBB2+, HR+ or HR−	9	22.0
TNBC	16	39.0
**Metastatic sites**		
Bone-only	3	7.3
Visceral-only	15	36.6
Bone and visceral	20	48.8
Others	3	7.3
**Number of prior lines of therapy**		
0	9	22.0
1	12	29.3
2	8	19.5
≥ 3	12	29.3
**Type of previous lines of therapy**		
Hormonal	2	4.9
Chemotherapy	19	46.3
Hormonal and chemotherapy	11	26.8
**Ki-67**		0.0
≤ 15%	4	13.8
16–30%	10	34.5
> 30%	15	51.7
**Tumor grade**		
G1	1	2.4
G2	21	51.2
G3	19	46.3

Bone-only metastases were present in three patients, making it the most frequent single-site of metastases (out of seven patients in total). Visceral-only metastases were detected in fifteen patients, and others (skin, brain) in three. Almost the half of the patients (n = 20) had a metastatic dissemination of both sites, bone and visceral metastases.

The majority of our patients had received at least one line of therapy before molecular profiling (78%), and almost a third (29.3%) received more than two lines. Previous therapies consisted in sequential chemo- and hormonal therapy; 43% of patients received chemotherapy in the first line and 4.9% a hormonal therapy in 4.9%.

**Figure 1:**
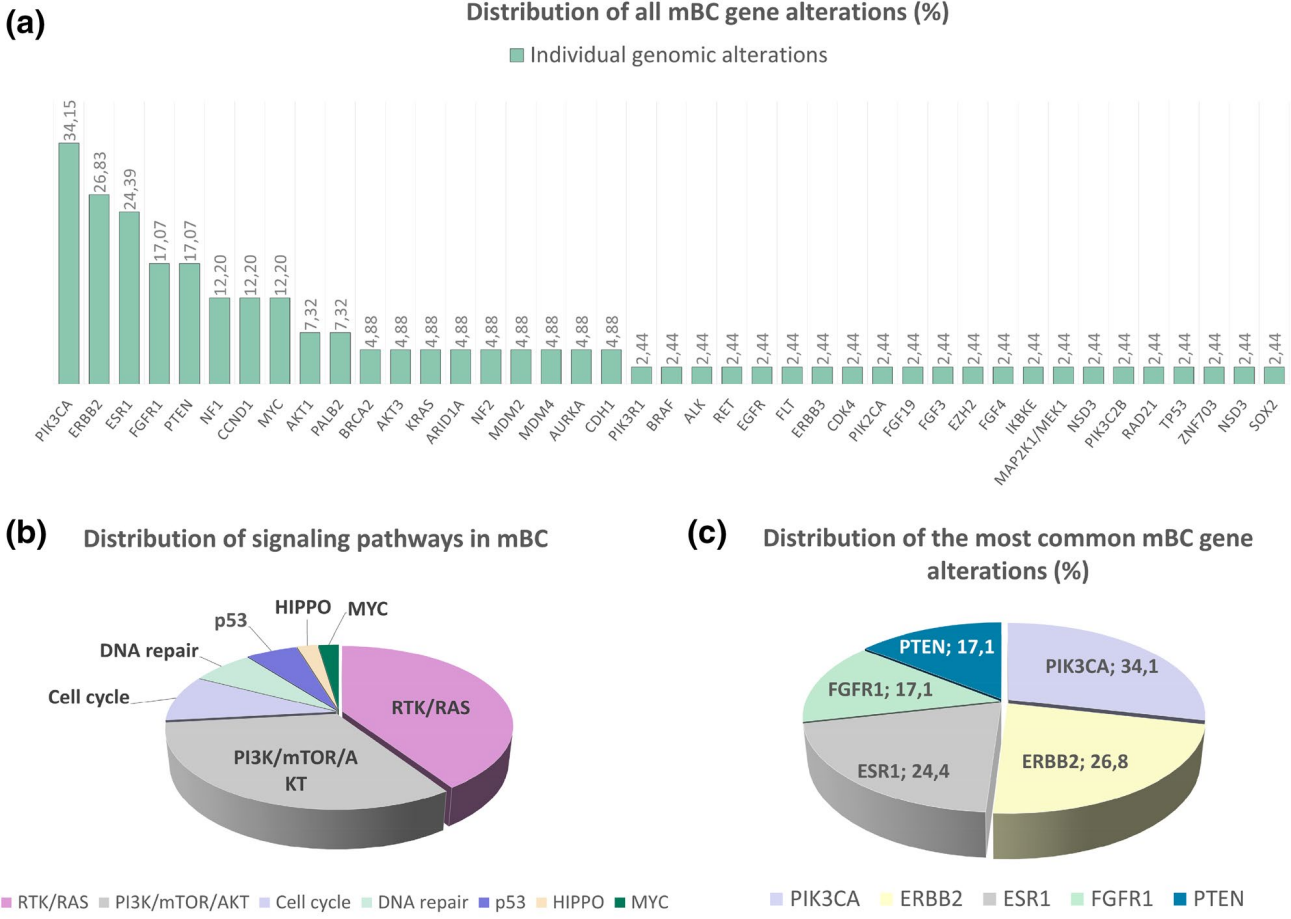
The results of NGS analysis of metastatic breast carcinoma samples, as detected by FoundationOne CDx. Distribution of detected mBC gene alterations (**a**), most commonly detected mBC gene alterations (**b**) and distribution of gene alterations in cancer signaling pathways (**c**).

Overall, 41 different genetic alterations were reported (Fig. 1a). MSI was detected by a single case (2.4%).

The prevalence of individual genetic alterations detected in our BC cohort (n = 41) was comparable to results obtained from a significantly larger database (n = 3,871)^14^, as summarized in Table 2. There was an average of 2.7 genomic alterations per metastatic patient. Most patients had more than one alteration (n = 27; 66%), and therefore more treatment options available. The most common alterations were *PIK3CA* (n = 14) and *ERBB2* (n = 11), followed by *ESR1* (n = 10), *FGFR1* (n = 7) and *PTEN* (n = 7), as shown by Fig. 1b.

**Table 2:** Comparison of individual genetic alterations prevalence among our and reference cohort^14^.

Alterations	Our data (%) n = 41	Reference data (%) n = 3,871
*PIK3CA*	34.9	31
*ERBB2*	26.8	33.3
*FGFR1*	17.1	12.3
*BRCA2*	4.9	4
*BRAF*	2.4	2.3
*EGFR*	2.4	3

The most frequently altered oncogenic signaling pathway in our cohort was RTK/RAS (81.4%), comprised of *ERBB2* in 33.3%, *FGFR1* in 21.2% and *NF1* in 15.2%. PI3K/mTOR/AKT mammalian target of rapamycin (mTOR) pathway alteration was detected in 65.8% of cases, most frequently by *PIK3CA* alteration (52%), followed by *PTEN* (26%) and *AKT1* (11%). Cell-cycle related pathway alterations were observed in 19.5% of cases, including alterations of *CCND1* (62.5%), *AURKA* (25%) and *CDK4* (12.5%). 14% of the patients had alterations in the DNA repair pathway with *BRCA1* and *PALB2* alterations. Alterations in p53 pathway were detected in 12.2% of patients; *MDM2* (40%), *MDM4* (40%), *TP53* (20%). At the same frequency of 12.2%, was an alteration in *MYC* oncogenic pathway, encompassing only an alteration in the *MYC* gene. At 4.8% of incidence, there was alteration in the HIPPO pathway (*NF2* alteration). The results are shown in Fig. 1c below.

We further classified our patient’s individual recurrent genomic alterations based on the strength of evidence from clinical studies, according to the ESMO Scale for Clinical Actionability of molecular Targets (ESCAT)^7,15^, as an aid of prioritizing the treatments. Table 3 provides a list of actionable and biologically relevant alterations detected in our cohort. Strictly focusing on the tier subset of clinically actionable genetic targets, 68.3% of patients harbored at least one such an alteration, (e.g. *PIK3CA* or *ERBB2*), and therefore belonged to tier I/tier II group, while 17% had a single-only actionable alteration. 12.2% of our patients belonged to tier III and 46.3% belonged to tier IV of ESCAT Classification. As of the genetic targets without evidence of clinical actionability, 25.6% of patients were belonging to the tier X subset, due to the presence of *CCND1* and *FGFR1* alterations.

**Table 3:** Our results classified based on ESCAT.

ESCAT	Readiness of use in clinical practice	Gene alteration	Total no.	Alteration prevalence (%)	Tier prevalence (%)
Tier I (I-A, I-B, I-C)	Targets are implemented in clinical routine decisions	*ERBB2* amplification	9	22.0	68.3
		*PIK3CA* mutation	14	34.1	
		MSI	1	2.4	
Tier II (II-A, II-B)	Investigational targets likely to define patients who benefit from a target drug, but additional data needed	*PTEN* loss	7	17.1	
		*AKT1* mutations	3	7.3	
		*ERBB2* mutations	2	4.9	
		*ESR1* mutations	10	24.4	
Tier III (III-A, III-B)	Clinical benefit previously demonstrated in other tumor type or for similar molecular targets	*BRCA 2* somatic mutation	2	4.9	12.2
		*ERBB3* mutations	1	2.4	
		*MDM2* amplification	2	4.9	
Tier IV (IV-A, IV-B)	Preclinical evidence of actionability	*NF1*	5	12.2	46.3
		*ARID1A*	2	4.9	
		*PALB2*	3	7.3	
		*CDH1*	2	4.9	
		*MYC*	5	12.2	
		*PIK3R1*	1	2.4	
		*TP53*	1	2.4	
Tier X	Lack of evidence	*CCND1* amplification	5	12.2	25.6
		*FGFR1* amplification	7	17.1	

In the clinics these NGS results were discussed at the multidisciplinary team meeting with the clinical case. The treatment options, the side effects and the patients quality of life were considered and in 31.7% the final treatment recommendations included the NGS findings.

### Relevant gene alterations for treatment

9/11 (82%) patients with *ERBB2* alteration showed an amplification by NGS and received an ERBB2 directed antibody therapy. Two patients showed an *ERBB2* mutation (L755S, A775_G776insYVMA), with treatment recommendation of osimertinib and afatinib or neratinib respectively.

8/14 (57%) patients with *PIK3CA* mutation were HR+/ERBB2−. The ERBB2 negativity of the tumor was detected by IHC or FISH and was confirmed by NGS for all eight patients. For this group of patients alectinib + fulvestrant was recommended. Three patients with *PIK3CA* mutation were TNBC. Ten patients harbored *ESR1* gene alteration, for which everolimus + anastrozole is recommended. As for *FGFR1* amplification*,* present in seven patients, aromatase inhibitors are recommended.

MSI was detected in one single case (2.4%). This patient was HR+/ERBB2− and progressive after endocrine and chemotherapy. Recommendation of MTB was pembrolizumab^16^.

The most common treatment option taken the information of molecular gene alterations and protein expression together was an ERBB2 based therapy followed by alpelisib in a combination with fulvestrant in postmenopausal and trastuzumab emtansine in premenopausal patients.

## Discussion

With the broad availability of DNA sequencing techniques, each patient can undergo the detection of the most common genetic alterations, which can be therapeutically targeted to achieve the best tumor response. To find the best treatment decision both the detection of potentially drugable gene alterations and information about protein expression has to be analyzed in the account.

The classical example of this approach is the recent approval of alpelisib^3^. *PIK3CA* mutations are identified in 40% of BC patients, mainly hormone receptor-positive and ERBB2−^4,17^. Recently in the SOLAR-1 trial the efficacy of the combination of alpelisib with fulvestrant in *PIK3CA* altered ER+ advanced BC was shown, and subsequently, the combination received the approval by FDA^18^. Therefore, this genomic alteration belongs to tier I of ESCAT classification, meaning that targets are implemented in clinical routine decisions. In our study, FoundationOne CDx could identify 14 patients (34%) who had *PIK3CA* mutation; but only 8 patients were HR+ and got the recommendation for alpelisib + fulvestrant. Three patients harbouring *PIK3CA* mutation were TNBC, and therefore, oral AKT inhibitor capivasertib was recommended to them^19,20^.

In our data an *ERBB2* amplification was found in nine patients with NGS compared with only seven ERBB2 positive cases analysed by IHC or FISH. It is necessary to find a threshold on basis of CNV which allows a better interpretation of NGS based amplification analysis. Two *ERBB2* mutations were detected in our cohort, namely A775_G776insYVMA insertion and L755S. ERBB2 A775_G776insYVMA results in a gain of function as well due to insertion of four amino acids in the protein kinase domain of the ERBB2 protein between amino acids 775 and 776. This results in an increased phosphorylation of ERBB2 and an activation of downstream signaling. Afatinib and neratinib were shown to be active for this mutation in vitro^21^. L755P and L755S are the most common ERBB2 mutations in mBC and leads to oncogenic transformation in cell culture assay. L755S mutation lies within the protein kinase domain and results in increased phosphorylation of ERBB2 and an activation of downstream signaling. L755S is associated with resistance to ERBB2 targeted therapy. Osimertinib demonstrated good efficacy against L755P and L755S mutations, the most common mutations in breast cancer^21^.

MSI is rare event in breast cancer, but patients with MSI mBC seems to benefit from pembrolizumab as recently reported^16^.

As an example of an alteration belonging to tier II, there is *ESR1*. Tumor cells with *ESR1* gene alteration are considered poor responders to aromatase inhibitors^22^. All *ESR1* mutations were found in the initial hormone receptor-positive tumors; possibly explaining the recurrence and helping clinicians to recommend more efficient anti-hormonal treatment (i.e. everolimus, temsirolimus) instead of changing the anti-hormonal treatment.

The *FGFR1* amplification is considered responsible for non-responding to tamoxifen^23^, and it was found in seven patients; categorized under tier X of ESCAT classification.

14% of the patients had alterations in the DNA repair pathway with *BRCA* and *PALB2* alterations. PARP inhibitors are just approved for germline mutations. Basket trials are necessary to better understand the role of *ERBB2* mutations, somatic alterations in DNA repair pathway and MSI in mBC.

Since these alterations are rare events, analysis of real-world data is necessary for an appropriate recruitment of basket trial. In consequence, the OnkoVision alliance was founded to develop an artificial intelligence (AI) based decision support platform to introduce molecular tumor boards in clinical routine. The platform should help to define treatment options for precision medicine based on factors such as druggable gene alterations, coexisting gene alterations, copy number alterations, MAF and protein expression.

Our NGS analysis prevented further antihormonal treatment for three patients and suggested the most promising treatment with tyrosine kinase inhibitors, due to the lack of evidence of targeted treatments.

Mutational evolution is a known phenomenon in cancer genetics. The change in the hormonal profile of breast cancers has been proved by studies in the past^24^. In the German national oncology guidelines^25^, a re-testing of every metastasis is recommended for hormone receptors and ERBB2 expression. All patients in our study underwent re-testing of hormonal profile with conventional techniques and those results were compared with the results of NGS. With tumour progress mutations change from the primary tumour to the metastasis. In most early stages the adjuvant therapy cures the patient from the cancer. The extensive phenotypic heterogeneity of breast cancer is currently researched extensively^26^. Clinically this would warrant a retesting of every metastasis as early as possible in the treatment lines. But clinical trials need to provide the evidence for such recommendations. Here our analysis adds to the clinical implementation data of NGS.

Fourteen of our patients (34.1%) had only one detectable alteration, regardless of whether actionable or non-actionable. This set of single-only detectable mutations harbored nine different mutations, underlining the clinical difficulty to recommend an effective treatment by conventional means. The use of NGS in the metastatic situation is beneficial for treatment decisions from our point of view. Because of the limited sample size and short duration of follow-up, assessment of progression-free survival and overall survival is too premature. However, our data shows how molecular analysis of advanced cancers could identify genomic alterations. This will identify possible drugable known cancer-driving pathways. Although our study comprised only 41 patients, the results are consistent with the prevalence of individual genetic alterations in BC, as compared to a larger database.

Now that we can individualize the treatment according to the tumor genomic alterations, the next step will be to prove its benefits for patients. Treatment options are more and more focused on genomic alterations, but not essentially approved across multiple tumor types, thus increasing the difficulty for clinicians to either treat the pathways or withhold treatment options as they are not approved within a given tumor type.

## Conclusion

The NGS of mBC supports the decision for the most promising treatment option in 58.5% of our patients with a Tier I evidence. For an appropriate treatment decision, it is necessary to evaluate gene alterations and protein expression of metastases in account. For an appropriate interpretation of rare gene alterations in mBC, basket trials on basis of real-world data are necessary. We founded the OnkoVision alliance to bring MTB into clinically routine and to identify appropriate patients for basket trials.

### Ethical approval

All procedures performed in studies involving human participants were in accordance with the ethical standards of the institutional and/or national research committee and with the 1964 Helsinki declaration and its later amendments or comparable ethical standards. Experimental protocols were approved by the institutional ethic committee of the Cancer Center Dachau.

### Informed consent

Informed consent was obtained from all individual participants included in the study.
